# Supplementary figures and images for: Infectome analysis of bat kidneys from Yunnan province, China, reveals novel henipaviruses related to Hendra and Nipah viruses and prevalent bacterial and eukaryotic microbes
Source: PLoS Pathog. 2025 Jun 24;21(6):e1013235. doi: 10.1371/journal.ppat.1013235 (PMC12187171; doi:10.1371/journal.ppat.1013235)

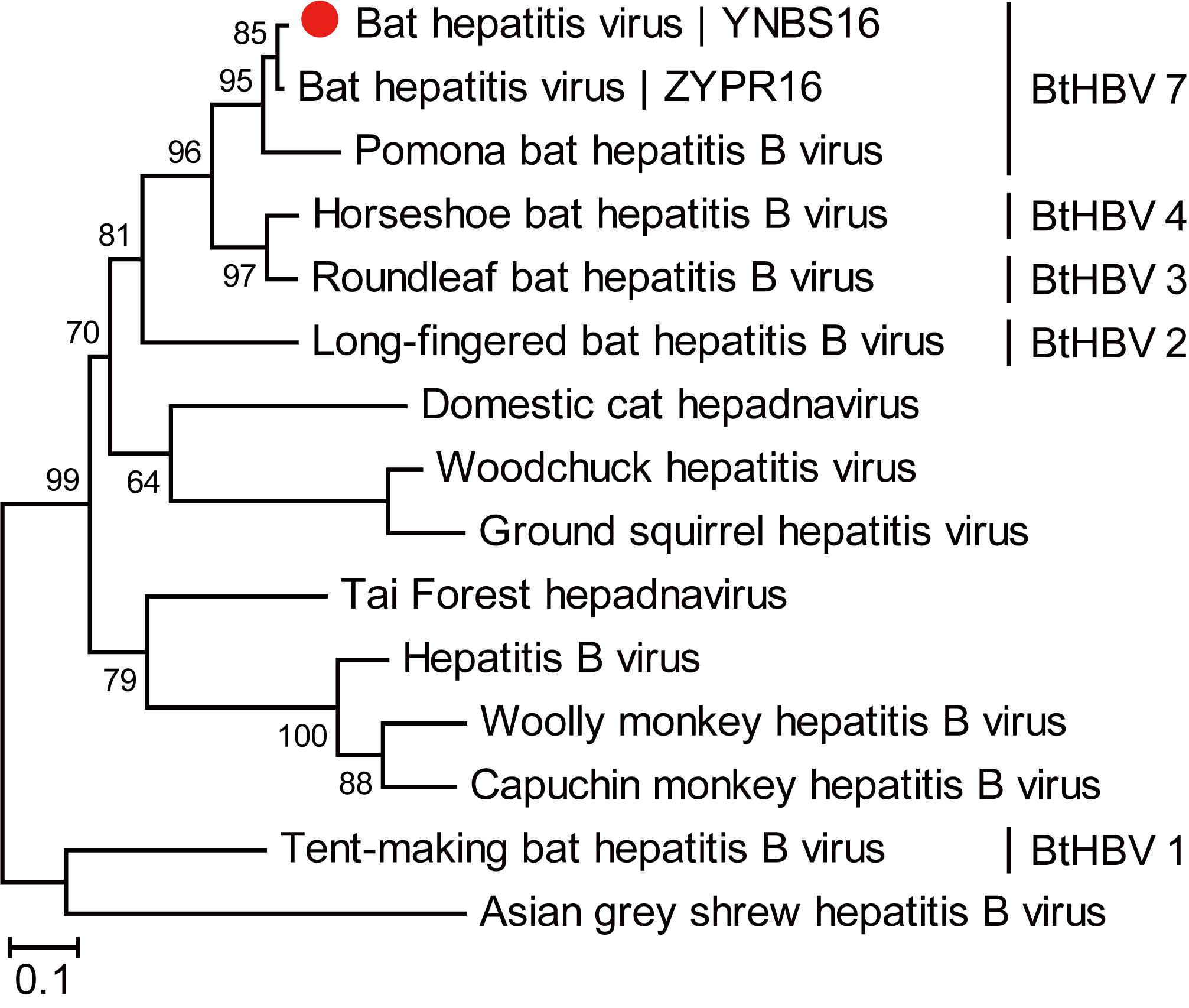

Supplement: S1 Fig — The newly identified virus in this study is marked with a solid red circle. Bat-derived viruses and their corresponding clades—determined according to divergence levels used by ICTV as species demarcation criteria—are labeled on the right. (TIF) [file ppat.1013235.s001.tif]
