# Supplementary material for: Infectome analysis of bat kidneys from Yunnan province, China, reveals novel henipaviruses related to Hendra and Nipah viruses and prevalent bacterial and eukaryotic microbes
Source: PLoS Pathog. 2025 Jun 24;21(6):e1013235. doi: 10.1371/journal.ppat.1013235 (PMC12187171; doi:10.1371/journal.ppat.1013235)
Supplement: Attachment — Submitted filename: Response to Reviewers.docx [file ppat.1013235.s006.docx]

**Response to Reviewers for manuscript PPATHOGENS-D-25-00320**

Title: Infectome analysis of bat kidneys from Yunnan province, China, reveals close relatives of Hendra-Nipah viruses and prevalent bacterial and eukaryotic pathogens

Dear Dr. Obbard, Dr. Swanstrom, and Editors,

We would like to express our gratitude for the constructive feedback provided by you and the reviewers. We have carefully addressed all comments, and the revised manuscript incorporates the suggested changes. Below, we provide a point-by-point response to the reviewers’ comments. Reviewer comments are presented in italics, followed by our responses in regular font.

In accordance with the journal’s requirements, we have also uploaded all main figures as separate files in the appropriate .tif formats. To ensure they meet the proper resolution and size, we used the PACE digital diagnostic tool for resizing and optimization.

We also revised the funding format to match the journal’s guidelines, and stated that the funders had no role in study design, data collection and analysis, decision to publish, or preparation of the manuscript.

Thank you for your time and consideration. We hope the revised manuscript meets the expectations of the reviewers and editors.

Best regards,

Guopeng Kuang, Edward C. Holmes, Mang Shi, and Yun Feng, on behalf of all authors窗体顶端

窗体底端

**Editorial Comments**

**Editor Comments:**

*Thank you for your submission. As you will see, all three reviewers agree on the value in this work, and feel that - overall - it is very well done. The manuscript is interesting, and relatively easy to read, and the novel henipavirus give the paper quite a broad level of interest.*

*In any revision, I ask that you take on board all of the reviewers’ comments. I suspect the vast majority of comments should be relatively easy to address through textual changes (e.g. more stringent use of the word 'pathogen') or through a change in framing. While I would welcome more detailed analysis of non-viral microbiota and any further validation that might be possible, given the scale of the manuscript as it stands I recognise that further work along these lines might be beyond the scope of what is feasible.*

**Response:**

We thank the editor for the constructive and supportive feedback. Following your advice, we have revised the manuscript thoroughly. In particular, we have used more cautious terminology when referring to microbes as “pathogens”.

The manuscript title has been revised, to read:

“Infectome analysis of bat kidneys from Yunnan province, China, reveals novel henipaviruses related to Hendra and Nipah viruses and prevalent bacterial and eukaryotic microbes”

Similarly, for example, Lines 99–101 have been revised to reads:

“…Herein, we utilized a meta-transcriptomics approach to investigate the total infectome—comprising viruses, bacteria, and eukaryotic microbes—in bat kidneys collected from this geographic region.”

We also fully agree that a more in-depth analysis of the non-viral microbiota—such as higher-resolution taxonomic profiling or experimental validation—would further strengthen the study. Hence, in response to Reviewer #2’s suggestion, we conducted additional analyses using BLASTx searches on contigs associated with marker genes, which yielded results consistent with our original findings. Although, as noted by Reviewer #1, further RNA/DNA sequencing of individual samples would indeed provide additional resolution, such efforts would require considerable time and financial resources, which are currently beyond the scope of this study.

**Editor Comment 1:**

*A clear statement on the methodology and nets used for trapping (mist nets, sticky nets?) and how the approach used conforms to the relevant ethical standards.*

**Response:**

We have revised the manuscript to clearly describe the bat trapping methodology and demonstrate compliance with relevant ethical standards. The sampling procedures were approved by the Ethics Committee of the Yunnan Institute of Endemic Disease Control and Prevention (File No. 20160002). The revised manuscript now includes the following updated text:

“…From 2017 to 2021, bats were captured using mist nets (12 m × 2.5 m, mesh size 38 mm) deployed near caves and orchards during evening hours. To minimize stress, nets were inspected every 15–20 minutes by trained personnel. Only bats displaying signs of weakened vital functions were selected for dissection. They were first humanely euthanized via intracardiac injection of sodium pentobarbitone and subsequently dissected for internal organ collection, following approved ethical protocols.”

**Editor Comment 2:**

*Full availability of all raw and processed sequences under PRJNA1184956 - which is not yet publicly available.*

**Response:**

We have made the project PRJNA1184956 publicly available at the NCBI SRA (<https://www.ncbi.nlm.nih.gov/bioproject/PRJNA1184956>), and the NCIBI/GenBank accession numbers (PQ621837 to PQ621860, PQ815815, and PQ824231) corresponding to the consensus viral genomes generated in this study have also been made publicly available.

**Part I – Summary**

**Reviewer #1:**

*The manuscript by Kuang et al. investigates the total infectome present in bat kidneys collected from Yunnan, identifying close relatives of highly significant pathogens, including henipaviruses—BSL-4 level viruses—alongside abundant bacteria and parasites. While the study is limited in scale in terms of the number of animals and geographic coverage, its findings are highly valuable and of great interest to the virology community. Notably, the discovery of bat-associated henipaviruses is both novel and significant. Previous studies were either based solely on serological evidence or lacked reliability due to the absence of PCR confirmation. Another key strength of this work is its novel approach in characterizing the total infectome, rather than focusing solely on virome or specific viral families like coronaviruses, providing a more comprehensive perspective.*

**Response:**

We sincerely thank the reviewer for the positive and insightful comments. We appreciate the recognition of the novelty and significance of our identification of bat-associated henipaviruses. Regarding the noted limitations in sample size and geographic coverage, we fully agree. These constraints primarily reflect the practical limitations on the number of individuals that can be ethically captured and dissected.

**Reviewer #2:**

*This article presents interesting data on the infectome of the kidneys of 10 different bat species in five regions of Yunnan Province in China (Fig1). New viruses, including some with zoonotic potential (henipaviruses), as well as a novel protozoan and 2 bacteria, were identified using a meta-transcriptomic approach (Fig2 and 3). The two henipaviruses were subsequently confirmed by qRT-PCR and nested RT-PCR testing of different tissues to determine virus distribution within 2 bats, one testing positive in the lung and kidney and the other testing positive in all tissues (heart, liver, kidney, gut) except the brain. The newly identified henipavirus 1 was present in 1 of the 20 libraries, and the henipavirus 2 was present in 2 of the 20 libraries, both corresponding to Rousettus leschenaultii and collected near areas with fruit orchards. Individual bat testing identified 4 bats carrying these henipaviruses, and within these bats, several tissues tested positive for the virus with the kidney showing the highest sequence yield (supplemental material), suggesting this is the target tissue tropism for these viruses. Characterization of the ORFs, identified the sequences for the 6 proteins found in henipaviruses, and phylogenetic analysis grouped these new viruses with other bat henipaviruses, including the zoonotic Hendra and Nipah. Observation of variable clustering depending on which of the 6 genes was being evaluated suggests recombination events (Fig4). The 2 novel bacterial species described include a Flavobacterium (94% nucleic acid identity in the ropB gene) and a Phyllobacterium (97% nucleic acid identity in the groEL gene) (Fig 5). The rationale for the selection of these genes was unclear, and the relevance of these findings was only briefly mentioned. It was noted that bacteria such as Leptospira were expected to be found in the kidneys of these bats, but weren’t. The protozoan from the Klossiellidae (Fig6) was present in 6 pools, and analysis of the COX1 and the cytB genes resulted in high similarity to Klosiella equi, an organism not usually considered pathogenic. The bat species identification was done by morphological characteristics and confirmed by genetic testing of COX1. Bats were pooled into 20 groups with variable numbers (2-8 individuals) for sequencing library construction. The authors acknowledge the drawback of sample pooling (lines 264 and 290) but also demonstrate the value of this approach. Two of the 20 libraries rendered no microbial presence. Library preparation, sequence curation, and phylogenetic analysis protocols described in the materials and methods seemed appropriate. The results of this study provide valuable information: The results indicated a preponderance of RNA viruses (20 species within 12 families), one DNA virus, one reverse-transcribing virus, one protozoan, and two abundant bacterial species (one of them present in 65% of the libraries). Nine of the virus species were phylogenetically related to known mammalian viruses. The methodology is consistent with other studies, the manuscript is well-organized and written, and the figures are clear.*

**Response:**

We agree that additional clarification was needed regarding the selection of *rpoB* and *groEL* in our bacterial analysis. These genes are both highly conserved and abundantly expressed, making them consistently recoverable with sufficient coverage in our data set. Furthermore, they are widely recognized as reliable, protein-coding phylogenetic markers in bacterial systematics due to their high resolution at species level. We have clarified this rationale in the revised Methods section. The updated text now reads:

“…From the aligned reads, we generated consensus sequences for well-covered protein-coding regions, with a focus on phylogenetically informative loci such as *rpoB* and *groEL*, which provide high species-level resolution in bacterial systematics.”

To strengthen the relevance of our findings, we have expanded the Results section to include additional details on coverage and read depth The revised text now reads:

“…Both taxa were represented by assembled contigs with high sequence coverage and depth, enabling the confident reconstruction of marker genes for phylogenetic analysis. Specifically, the representative *rpoB* gene of *Flavobacterium* was assembled from group YNBS16, with a mean coverage of 76.8% and an average depth of 3.30X, while the representative *groEL* gene of *Phyllobacterium* was recovered from group YNBS01, with 91.9% coverage and a mean depth of 5.29X.”

These observations have also been addressed in the Discussion section, which now reads:

“…Although these bacteria were relatively abundant, their biological significance in bats remains unclear. On one hand, their high abundance in tissue may suggest a pathogenic role. Alternatively, they may represent commensals or opportunistic colonizers, although their presence in the kidney is less probable.”

Regarding the absence of *Leptospira* in our data set, we appreciate the reviewer’s insightful observation and acknowledge that this result was unexpected based on previous literature and our prior work. We have already noted in the manuscript that sample pooling may obscure low-abundance microbes:

“…Previous research identified *Leptospira spp.* in bat kidney, supporting the hypothesis that bat kidneys may serve as a reservoir for zoonotic *Leptospira* [28,29,46,47], and we previously detected pathogenic *Leptospira* in bat kidneys using nested PCR in individual tissue samples [48]. However, no *Leptospira*-associated reads were detected in the meta-transcriptomic sequencing of this study, possibly due to sample pooling which might obscure the detection of low-abundance microbes.”

To clarify this further, we have now updated the Limitations section to address potential reasons for the absence of *Leptospira* detection in this study. The revised text reads as follows:

“…Pooling may have reduced sensitivity for detecting low-abundance microbes and potentially compromised the accuracy of microbial quantification.”

We thank the reviewer for pointing out that *Klossiella equi* is not typically considered pathogenic. To better reflect current understanding, we have revised the wording in the Results section, replacing “protozoan pathogen” with “protozoan microbe”. The updated sentence now reads:

“Analysis of the *cox1* and cytochrome b (*cytB*) genes identified a protozoan microbe closely related to *Klossiella equi* of the family Klossiellidae (phylum Apicomplexa), which is known to infect the kidneys of horses [36].”

Finally, we appreciate the reviewer’s thoughtful acknowledgment of the strength and limitations of our sample pooling strategy. While pooling reduces resolution at the individual level, it allowed for efficient, large-scale screening of microbial diversity across multiple bat species and sampling locations. We have further highlighted both the strengths and limitations of this approach in the revised manuscript:

“…Although practical for broad surveys, pooling precludes resolving whether microbes originated from co-infections in single bats or from distinct individuals.”

**Reviewer #3:**

*Kuang and colleagues have investigated and reported the infectome of bat kidneys sampled in Yunnan Province, China. This manuscript is both timely and relevant, as it provides further evidence of novel microorganisms that infect wild animals, demonstrating specific tissue tropism and tissue specific viromes. The findings are particularly significant due to the identification of a divergent paramyxovirus that clusters within the same clade as lethal human pathogens such as Nipah and Hendra viruses. However, while the results are robust, the authors tend to overstate the pathogenic potential of the microorganisms identified. Throughout the manuscript, the microorganisms are frequently referred to as pathogens, despite the absence of experimental or indirect evidence—such as observed disease signs in animals—to support this classification. Follow additional comments below.*

**Response:**

We appreciate the reviewer’s thoughtful feedback and agree that the term 'pathogen' should be used cautiously without supporting evidence. We have revised the manuscript accordingly, replacing it with neutral alternatives and refining the framing to avoid overstating potential pathogenicity.

Specifically, we have updated the manuscript title to:

“Infectome analysis of bat kidneys from Yunnan province, China, reveals novel henipaviruses related to Hendra and Nipah viruses and prevalent bacterial and eukaryotic microbes”

Additionally, we revised the subtitle in the Results section to:

“Identification and characterization of abundant bacteria in bat kidneys”

For example, Lines 99–101 now read:

“…Herein, we utilized a meta-transcriptomic approach to investigate the total infectome—comprising viruses, bacteria, and eukaryotic microbes—in bat kidneys collected from this geographic region.”

Similarly, Lines 221–223 have been modified to:

“Analysis of the *cox1* and cytochrome b (*cytB*) genes identified a protozoan microbe closely related to *Klossiella equi* of the family Klossiellidae (phylum Apicomplexa), known to infect the kidneys of horses [36].”

**Part II – Major Issues: Key Experiments Required for Acceptance**

**Reviewer #1 Major Comment 1:**

*The authors should reference the recent publication in Nature Microbiology by He et al., which also reports the discovery of Nipavirus-like viruses in China. Although that study only identified viral fragments, and the authors of this manuscript failed to obtain longer genomic sequences using PCR, some discussion comparing the findings would be valuable.*

**Response:**

We thank the reviewer for this important comment. In the revised Discussion section, we have cited the recent study reporting a “compendium” of 8,176 bat RNA viral metagenomes (*Nature Microbiology*, 2025) and compared these data to our findings. While that study detected Nipah virus-like fragments in bats across China through metatranscriptomic sequencing of predominantly intestinal samples, it was limited to short viral fragments and did not recover full-length genomes. Nor was there any PCR confirmation. In contrast, our study successfully assembled two complete, full-length genomes of novel henipaviruses from bat kidney tissues, with additional qRT-PCR verification across multiple organs. Our results therefore provide detailed genomic and tissue-specific perspectives on henipavirus infection in bats, building upon and expanding prior indications of henipavirus circulation risk in China. The updated text in the revised manuscript reads as follows:

“…In this study, we identified two related henipaviruses in *Rousettus leschenaultii* bats, marking the first detection of full-length henipavirus genomes in bats from China.”

And also in the discussion section:

“…A recent large-scale study of bat RNA viral metagenomes detected genomic fragments of Nipah virus in bats from Southwest China [43], highlighting the potential circulation risk of Nipah-like viruses. However, these findings were not confirmed by RT-PCR, and the absence of full-length genomes further underscores the importance of our study, which provides genome-scale evidence for the diversity of henipaviruses in China and their zoonotic risk.”

**Reviewer #1 Major Comment 2:**

*The authors have done a thorough job in characterizing henipaviruses, making full use of the available data. However, the characterization of bacteria is relatively limited. While I understand that these may be of lesser priority, it would be valuable to perform single-library RNA and DNA sequencing on the samples with highest abundance of bacteria to obtain draft genomes. Any novel pathogens would merit revealing full genomes.*

**Response:**

While we agree that deeper characterization through RNA and DNA sequencing of individual libraries would strengthen the study, such efforts would require considerable additional resources and are currently beyond the feasible scope of this work.

**Reviewer #1 Major Comment 3:**

*Similarly, the transcriptomic characterization of parasites is currently limited to mitochondrial genes—what about nuclear genes? Expanding this analysis would provide a more comprehensive understanding of the parasite component in the infectome.*

**Response:**

We agree with the reviewer and have conducted additional analyses based on nuclear marker genes (e.g., *EF1-alpha*). However, we were only able to assemble sequences corresponding to the bat host, and did not recover *EF1-alpha* sequences from potential parasitic organisms. This may reflect the limited representation of *EF1-alpha* genes from phylogenetically related parasitic taxa in current reference databases, rather than their true absence in the samples.

**Reviewer #2**

**Reviewer #2 Major Comment 1:**

*Some clarification on the nets used for trapping is necessary. Can the type of net and source be provided?*

**Response:**

We agree with the reviewer and have revised the Methods section to include a detailed description of the mist nets, as follows:

“…bats were captured using mist nets (12 m × 2.5 m, mesh size 38 mm) deployed near caves and orchards during evening hours. To minimize stress, nets were inspected every 15–20 minutes by trained personnel.”

**Reviewer #3**

**Reviewer #3 Major Comment 1:**

*Page 4 line 57 - The authors stated “broader pathogen surveillance beyond the gastrointestinal tract” A pathogen per definition is a microorganism that induce disease. The authors have no data to infer pathogenicity even for the new henipaviruses. I recommend the authors to moderate their claims throughout the manuscript.*

**Response:**

We agree that the term “pathogen” should be used cautiously, especially in the absence of direct evidence of pathogenicity. In response, we have carefully revised the manuscript to moderate our claims and to use more neutral terminology. Specifically, the sentence on page 4, lines 57-59 has been revised to read:

“…Overall, these findings provide valuable insights into the infectome of the bat kidney, highlighting the need for broader microbial surveillance beyond the gastrointestinal tract.”

**Reviewer #3 Major Comment 2:**

*PRJNA1184956 - I could not find the raw dataset at NCBI SRA. Neither the consensus genomic data with the accession numbers provided.*

**Response:**

We thank the reviewer for bringing this to our attention. We have made the BioProject PRJNA1184956 publicly available at the NCBI SRA (<https://www.ncbi.nlm.nih.gov/bioproject/PRJNA1184956>). In addition, the consensus viral genome sequences generated in this study have also been deposited and are publicly accessible under the GenBank accession numbers PQ621837 to PQ621860, PQ815815, and PQ824231.

**Reviewer #3 Major Comment 3:**

*Page 16 line 352-360 - Does the authors consider that mapping reads to reference bacterial and fungi genomes can severely bias the results? For the taxa that are closely related to reference genomes it should be ok once reads will map against with high confidence, but for more diverging taxa reads will not even map since Bowtie 2 has a limited number of snps allowed to map a read against a reference sequence. As pointed out by the authors, there is no study investigating bat kidneys using metagenomics/metatranscriptomics. Therefore, most of the bacterial and fungi species likely infecting these bats may be substantially divergent from the reference genomes available in the databases. I recommend to the authors to take a more holistic view of bacteria and fungi performing Diamond search on the assembled contigs from Megahit and extracting the taxonomic identification from it.*

**Response:**

We agree with the reviewer that mapping-based approaches alone may overlook highly divergent microorganisms. To address this, we performed BLASTx searches targeting conserved bacterial marker genes (e.g., *rpoB*, *groEL*, *recA*, and *gyrB*) and eukaryotic microbial genes (e.g., *EF1-alpha*). These analyses yielded results consistent with our initial findings. We have also added the relevant information to the Results section, which now reads:

“Our meta-transcriptomic analysis revealed the presence of two relatively abundant bacterial taxa, *Flavobacterium* and *Phyllobacterium*, with conserved marker genes (*rpoB*, *groEL*, *recA*, and *gyrB*) identified via the BLASTx analysis of assembled contigs (Table S3)”

Moreover, our focus was on relatively abundant microbes, as low-abundance organisms with fragmented genomes were not prioritized in this study. The limited microbial diversity—aside from viruses—and the absence of highly abundant non-viral microbes are likely attributable to the nature of the tissue samples, which generally harbor fewer cellular microorganisms unless an active infection is present.

**Part III – Minor Issues: Editorial and Data Presentation Modifications**

**Reviewer #1:**

**Reviewer #1 Minor Comment 1:**

*Terms like “infectome” should be defined in the abstract as well.*

**Response:**

We thank the reviewer for this helpful suggestion. In response, we have revised the Abstract to define the term “infectome” upon its first mention. The updated text now reads:

“…However, the composition of the infectome (i.e., the collection of viral, bacterial and eukaryotic microorganisms) within bat kidneys remains poorly understood”

**Reviewer #1 Minor Comment 2:**

*The geographic locations and ecological factors (in any) of the newly found viruses should be discussed.*

**Response:**

We thank the reviewer for this insightful comment. We agree that discussing the geographic locations and ecological factors is essential for understanding the emergence and transmission risks of newly discovered viruses. In our study, the detection of two novel henipaviruses in *Rousettus leschenaultii* bats is particularly significant given that Yunnan province is a hotspot for bat diversity and is located in southwestern China, representing the region of China geographically closest to Malaysia, where NiV first emerged. We have incorporated this discussion in the revised manuscript to highlight the significance of Yunnan as a region with heightened zoonotic risk for henipaviruses. The updated text now reads:

“…This finding is particularly significant as Yunnan province is a recognized hotspot for bat diversity [31-34] and is located in southwestern China, representing the region of China geographically closest to Malaysia, where NiV first emerged (Fig 1A)”

**Reviewer #2:**

**Reviewer #2 Minor Comment 1:**

The references are adequate, but there are at least 3 references that are repeated (ref 21 is a repeat of 19, 49 is a repeat of 29, and 50 is a repeat of 30).

**Response:**

We apologize for the oversight regarding repeated references. In addition to the duplications pointed out by the reviewer (references 21 and 19, 49 and 29, 50 and 30), we also identified another duplicated reference (references 13 and 36) upon careful re-examination of the reference list. We have now thoroughly reviewed and revised the entire reference list and manuscript, removed all duplicated entries, and ensured that all in-text citations are accurate and consistent.

**Reviewer #3:**

**Reviewer #3 Minor Comment 1:**

*How the authors can ascertain if the protozoan and bacteria found are relevant for infection/pathogenicity, maybe they are just highly prevalent non deleterious symbionts*

**Response:**
We agree that the presence of protozoan and bacterial taxa in bat kidneys does not necessarily imply pathogenicity, and that some of these microorganisms may indeed represent non-pathogenic symbionts. We have revised the Discussion to more cautiously interpret the relevance of these microbes. The updated text now reads:

“Although these bacteria were relatively abundant, their biological significance in bats remains unclear. On one hand, their high abundance in tissue may suggest a pathogenic role. Alternatively, they may represent commensals or opportunistic colonizers, although their presence in the kidney is less probable.”

**Reviewer #3 Minor Comment 2:**

*Page 5 line 79-80 - The authors stated “While much of this research has focused on the bat gut virome, viruses present in other body sites—such as the kidneys—also pose transmission risks.” Those viruses present in the kidney are not supposed to be excreted through urine? That is the way Nipah is excreted and gets in contact with humans through a natural beverage contaminated with urine carrying infectious Nipah viral particles, for instance. One thing that may impact the chance of detecting the virus in the urine is that the viral particle is perhaps labile and degrades quickly and viral loads may not be that high compared with kidney tissue. An interesting strategy to sample wild bats and test this hypothesis which also can provide critical data to evaluate if the virus is being excreted is to sample bat urine and only after euthanasiate the bat to collect the kidney. But very good sampling procedures are required to avoid cross contamination of samples of the same bat in the field.*

**Response:**

We thank the reviewer for this thoughtful comment. We agree that viruses present in the kidneys are typically excreted through urine, and that human infection can occur through exposure to food or water contaminated with virus-laden urine, with Nipah virus serving as a well-established example of this transmission route. We have revised the sentence in the Introduction section to clarify the potential urinary excretion of kidney-associated viruses. The updated text now reads:

“…Although most research has concentrated on the bat gut virome, viruses residing in other tissues, including the kidneys where they may be excreted via urine, also present potential transmission risks.”

Furthermore, we greatly appreciate the reviewer’s valuable suggestion regarding the strategy of simultaneously sampling both urine and kidney tissues. This approach has provided important insights for future study design. We have added the following statement to the Discussion section to emphasize the importance of future research efforts:

“…Furthermore, considering that kidney-associated pathogens, such as henipaviruses, may be shed through urine [39,40], future research should incorporate both kidney and urine sampling to comprehensively evaluate pathogen shedding and the associated transmission risk.”

**Reviewer #3 Minor Comment 3:**

*Figure 1 A - I think it would be worthwhile for the readers to see the Yunnan province contextualized within China and bordering countries. Besides, if the authors can also depict it including the geography and biome of the region it is interesting for the readers to understand bats species and populations residing in these environments and why they are more likely related to bat species and population of neighbouring countries and not to other Chinese provinces. Providing the context of neighbouring countries is particularly important because Nipah outbreaks have been reported in neighbouring countries.*

**Response:**

We thank the reviewer for this valuable suggestion. Accordingly, we have revised Figure 1A to better contextualize Yunnan province within the broader geographical framework of China and its neighboring countries, including Myanmar, Laos, Vietnam, Thailand, Cambodia, and Malaysia, where Nipah virus outbreaks have been reported. The updated figure highlights Yunnan’s location in relation to national and international borders, and reflects the region’s diverse geography and ecological settings. To enhance clarity, we have also revised the figure legend accordingly. The updated legend now reads:

“…Map showing the five sampling locations in Yunnan province, China, with nearby countries (Myanmar, Laos, Vietnam, Thailand, Cambodia, and Malaysia) shown for reference. Pie charts indicate the species composition of the bats sampled at each site.”

**Reviewer #3 Minor Comment 4:**

*Page 7 line 112 - “Based on mitochondrial sequences and sampling locations, the samples were pooled into 20 groups for” - Does it mean that samples from the same species and caves/sites were pooled together? What about samples of the same population from different time points or there were no resampling of the same population in different time points?*

**Response:**

Only at the “NJ” site were 23 individuals collected across different years (15 in 2017, 6 in 2018, and 2 in 2019) and pooled into three libraries. While these samples came from the same population at different time points, our study does not examine temporal dynamics. Pooling was done to increase sample size and improve detection sensitivity. As our focus is on overall infectome diversity, we believe this does not affect the validity of our findings.

**Reviewer #3 Minor Comment 5:**

*Figure 2 B - This is a very difficult graph to interpret. I suggest the authors look for an alternative way to show these results. In fact, these results are already shown in much more detail in section C. It is not the same metric, but the presence of viruses per taxonomic level distributed per host species is already there in much more detail.*

**Response:**

We have revised Figure 2B to improve its clarity and interpretability. Specifically, we added a clear label within the figure indicating “Number of species detected for each microbial type”. Additionally, we updated the figure legend to include the statement “Number of viral, bacterial, and eukaryotic microbial species detected, with color schemes corresponding to those used in panel C.” ensuring visual consistency between panels B and C. We believe these revisions enhance the clarity and coherence of the figure, making the presentation of results more accessible to readers.

**Reviewer #3 Minor Comment 6:**

*Figure 2 B- How the authors determined that, for instance, Yannan bat henipavirus 2 what was present in different pools of H. armiger? Did these henipaviruses show high sequence identity? I have not seen a methodological description of how the authors approached the detection of the same virus in multiple samples in the material and methods. The only section about this regards the use of ICTV parameters, but it would be important to provide more detail stating for instance, that “we considered the same virus present in multiple samples when ???? clustered together in the phylogenetic tree and showed aa or nucl identity of ??? in the entire genome??”*

**Response:**

We apologize for the confusion. Virus identification was performed using all contigs from all libraries, with similar contigs grouped into species according to ICTV species demarcation criteria. These species-level sequences were then used as reference templates to map reads from each individual library, allowing us to determine which viruses are present in each library. We have revised the relevant text for clarity, now reading:

“…To assign species-level classifications, all viral contigs were clustered using CD-HIT (v4.8.1) [59], applying identity thresholds based on ICTV species demarcation criteria for the corresponding viral genera [55]. Representative contigs from each cluster were then compared to known viral species to determine whether they matched previously recognized or potentially novel viruses.”

“… Reads mapped to all contigs assigned to the same viral species were aggregated to calculate the final abundance of that species in each library.”

**Reviewer #3 Minor Comment 7:**

*It is interesting that the authors found Chuvirus and other viruses in the kidney tissue of several different bat species and they did not comment on the possibility of these viruses to infect mammals, even though the current knowledge suggests that these viruses have other animals taxa (invertebrates etc) as main hosts. Finding a full genome of a virus within the kidney suggests that the virus may be infecting that tissue. On the other hand, it is hard to associate the presence of such viruses as of dietary sources as pointed out in page 8 line 154-155. Except for the Toti and Narna comments by the authors on Page 11 line 217.223.*

**Response:**

We thank the reviewer for this important comment. We agree that the detection of full genomes of Chuviruses and other viruses, traditionally considered to have invertebrates as their primary hosts, in bat kidney tissues suggests the potential for active infection rather than incidental dietary contamination. Indeed, recent studies have increasingly shown that many RNA viruses previously thought to be restricted to invertebrates, including chuviruses, are also found in wild mammals, often with multi-organ distribution and across multiple host species, indicating that these mammals may serve as bona fide hosts rather than passive carriers (Chen et al., 2023, *Cell*). We have revised the Discussion section accordingly to address this important point. The updated text now reads:

“…Furthermore, we detected near-complete genomes of chuviruses in bat kidney tissues, which questions their usual assignment as viruses that do not infect vertebrates. While it is possible that these viruses infect eukaryotic microbes within the host, recent studies have increasingly identified Chuviruses in mammals [14], suggesting that their host range may be broader than their previous designation as insect-specific viruses”

**Reviewer #3 Minor Comment 8:**

*Page 8 line 142-145 - amino acid or nucleotide identity? L protein I assume amino acid. This is a substantial difference.*

**Response:**

We agree that it is important to clarify the type of sequence identity reported. Hence, we have carefully revised the text to specify that the identity percentages refer to amino acid sequence comparisons of the L protein. The revised sentence now reads:

“…Notably, the two newly identified henipaviruses showed relatively close evolutionary links to two human pathogens—Hendra virus (HeV, 52.23–56.94% amino acid identity in the L protein) and Nipah virus (NiV, 52.17–57.03% amino acid identity).”

**Reviewer #3 Minor Comment 9:**

*Page 10 line 193 - “likely reflects the action of recombination.“ or due to the high divergence it may represent the accumulation of several mutations leading to the phylogenetic signal saturation leaving few informative sites that allows more consistent phylogenetic grouping inferences. Please, add alternative explanations even more considering that the data is not suitable to evaluate recombination (divergent viruses).*

**Response:**

This is a fair point. We agree that, in addition to the possibility of recombination, the observed topological variability could also be explained by high sequence divergence leading to the accumulation of mutations and resulting in phylogenetic signal saturation. This process may reduce the number of informative sites available for robust phylogenetic inference. In response, we have therefore revised the relevant sentence in the Results section to incorporate this alternative explanation and clarified that the current data are not optimal for formally assessing recombination in such divergent viral sequences. The updated text now reads:

“…Alternatively, the topological inconsistency may result from high sequence divergence, leading to substitution saturation, increased phylogenetic noise, and hence fewer informative sites for reliable inference”

**Reviewer #3 Minor Comment 10:**

*Page 10 line 197 - “bacterial pathogens” How the authors know that these are pathogenic bacteria, this same question also applies to viruses and fungi found? Were there any signs of disease in the sampled bats? Although there is some indirect evidence that some may be pathogens (belonging to a family/taxa that include several known pathogens and are phylogenetically related to known pathogens) there is no other supporting evidence to suggest pathogenicity in this manuscript.*

**Response:**

We thank the reviewer once again for this thoughtful and important comment. As noted in our responses to earlier comments in **Summary Comments (Part I)**, we have carefully revised the manuscript—including the title, abstract, and main text—to ensure that terminology related to pathogenicity is used with appropriate caution. Specifically, we have replaced terms such as “pathogens” with more neutral alternatives like “microorganisms” or “microbes” throughout the text, unless there is direct or well-supported evidence indicating a disease association. This adjustment reflects the current limitations in our data, as we did not observe clinical signs of disease in the sampled bats, nor do we present functional or pathological validation of pathogenicity in this study.

**Reviewer #3 Minor Comment 11:**

*Page 12 line 239 - “our study identified close relatives”. Close relatives is a bit strong and arbitrary adjective based on the amino acid divergence and instability of the phylogenetic positioning depending on the protein used for phylogenetic reconstruction. I suggest the authors moderate it. The results are important and interesting, but those are not close relatives of Hendra and Nipah.*

**Response:**

We agree that the phrase “close relatives” may imply a stronger evolutionary relationship than is supported by the available data, especially considering the observed amino acid divergence and variability in phylogenetic placement depending on the protein used. To ensure greater precision and avoid overinterpretation, we have revised the sentence as follows:

“…Of particular note, our study identified two novel henipaviruses that cluster within the bat-associated clade of the genus**,** including the lineage containing Hendra and Nipah viruses.”

In addition, we have updated the manuscript title to more accurately reflect the findings without overstating phylogenetic proximity:

“Infectome analysis of bat kidneys from Yunnan province, China, reveals novel henipaviruses related to Hendra and Nipah viruses and prevalent bacterial and eukaryotic microorganisms”

**Reviewer #3 Minor Comment 12:**

*Page 12 line 246 - italicize Pteropus*

**Response:**

Corrected as suggested.

**Reviewer #3 Minor Comment 13:**

*Page 13 line 278-9 - “The pathogenicity of these eukaryotic parasites to humans or even bats remains unclear.” Here the authors emphasized that more studies are necessary to ascertain if the identified microbes are pathogenic. But I think the authors must review more throughout the wording and tone throughout the manuscript as pointed out multiple times above including in the title.*

**Response:**

As noted in our response to the **Summary Comments (Part I)**, we have undertaken a thorough and systematic revision of the manuscript, including the title, abstract, and main text, to ensure the cautious use of the term “pathogen”. In particular, terms that could imply pathogenicity have been replaced with neutral descriptors such as “microorganisms” or “microbes” unless supported by direct evidence of disease association.

Regarding the specific statement “The pathogenicity of these eukaryotic parasites to humans or even bats remains unclear”, we have retained this phrasing to accurately reflect the current uncertainty, and have ensured that similar cautious wording is consistently applied across the manuscript.
